# Editorial: Effects of human activities on microorganisms and microbial carbon cycle in coastal waters

**DOI:** 10.3389/fmicb.2025.1594821

**Published:** 2025-04-08

**Authors:** Yaodong He, Mohan Bai

**Affiliations:** ^1^School of Fisheries, Zhejiang Ocean University, Zhoushan, China; ^2^Agro-Environmental Protection Institute, Chinese Academy of Agricultural Sciences, Tianjin, China

**Keywords:** human activities, microorganisms, carbon cycle, coastal water, pollutant, microbial community

Coastal regions are home to microbial communities and carbon cycles that are vital to human survival and marine health. However, human activities are disrupting this delicate balance in unprecedented ways. Industrial pollutants such as heavy metals and oil spills directly interfere with microbial photosynthesis and organic matter decomposition—critical processes that underpin the carbon cycle. Aquaculture introduces excess nutrients from fish excreta and residual feed into coastal waters, triggering algal blooms and exacerbating oxygen depletion, which destabilizes the balance of carbon storage and release. Overfishing further disrupts natural regulation by reducing fish populations that help break down organic matter, thereby altering the transfer of carbon to the atmosphere or sediments. Additionally, tourism-related activities risk introducing non-native microbial species that can outcompete indigenous communities and compromise the stability of the carbon cycle. These cumulative anthropogenic pressures pose systemic risks to coastal microbial ecosystems, requiring urgent scientific efforts to clarify their underlying mechanisms and cascading environmental impacts. Addressing these challenges requires global collaboration and interdisciplinary innovation. Advanced genomic tools and ecological modeling must be leveraged to decode microbial responses to pollution, eutrophication, and other stressors and to inform targeted management strategies. For instance, the development of integrated multi-trophic aquaculture systems could mitigate nutrient overload, while stricter pollutant controls and fishery restoration programs could revive synergistic interactions between microorganisms and macroorganisms. Cross-border biosecurity protocols are also essential to contain invasive species and preserve native microbial functions. Protecting these invisible “carbon engineers” is not only an ecological imperative but a lifeline for civilization itself, requiring collective action to harmonize human progress with the rhythms of coastal ecosystems.

This Research Topic “*Effects of human activities on microorganisms and microbial carbon cycle in coastal waters*,” which includes four original research articles and one review article, focuses mainly on microbial communities and the microbial carbon cycle in coastal waters affected by human activities. The research article by Hunt and Johnson presented results from the Piver's Island Coastal Observatory (PICO) time-series, which offers a critical insight into the dynamics of a mesotrophic coastal ecosystem in a biogeochemically dynamic and biodiversity-rich region under intensifying global change pressures. Spanning a decade of high-resolution observations, this dataset captures both abrupt perturbations and gradual, persistent shifts within the ecosystem. These longitudinal findings provide a decade of observations focusing on pulse and pressure ecosystem changes. The research article by Deng et al. provided us with an insight into the seagrass-associated microbes that are vital to seagrass health. Nutrient pollution reduces the biodiversity and functional complexity of leaf-surface (phyllosphere) microbial communities, accelerating seagrass decline by impairing critical microbial services. Another research article by Wang et al. also reported on seasonal and anthropogenic influences on bacterioplankton communities. Coastal bacterioplankton mediate critical biogeochemical cycles, but their structural and functional responses to seasonal and anthropogenic drivers remain poorly quantified. This study tracked bacterioplankton communities across seasons in estuaries in northern China, revealing distinct seasonal assembly patterns. Seasonal shifts in temperature, light, and nutrients drove these transitions. Summer tourism and riverine inputs increased nutrients, favoring photosynthetic taxa associated with phytoplankton blooms. Metabolic predictions highlighted seasonal adaptations—spring carbon fixation aligned with rising light/temperature, summer glycolysis supported high biomass energy demands, and autumn amino acid metabolism accelerated organic matter turnover. These findings underscore how seasonal dynamics and human activities are reshaping coastal microbial ecosystems through altered nutrient regimes, providing critical insights into microbial responses to global change pressures.

Human activities are fundamentally reshaping coastal ecosystems, with microbial communities emerging as both sentinels and architects of ecological resilience. As linchpins of marine carbon, nitrogen, and phosphorus cycles, microorganisms exhibit acute sensitivity to climate change (warming, salinity shifts, acidification) and anthropogenic pressures (eutrophication, pollution, aquaculture, tourism). Key studies reveal cascading impacts: eutrophication-driven biodiversity loss in seagrass phyllosphere microbiomes exacerbates host decline; freshwater inputs and dissolved inorganic phosphorus trigger delayed phytoplankton blooms dominated by opportunistic genera; seasonal nutrient fluctuations reorganize bacterioplankton metabolic strategies (carbon fixation in spring, glycolysis in summer, organic decomposition in autumn). At the same time, the environmental adaptability of pathogens such as Labyrinthula underscores the complex host-microbe-environment interactions that can tip ecosystems toward collapse or recovery. Taken together, these findings position microbes not just as passive indicators but as active mediators of ecosystem trajectories. In the future, integrating high-resolution microbial monitoring with multifactorial models will be critical for coastal management—recognizing that safeguarding macroscopic marine health in the Anthropocene requires decoding the invisible microbial networks that govern Earth's biogeochemical pulse.

